# Reduced Glutamate Turnover in the Putamen Is Linked With Automatic Habits in Human Cocaine Addiction

**DOI:** 10.1016/j.biopsych.2020.12.009

**Published:** 2021-05-15

**Authors:** Karen D. Ersche, Tsen Vei Lim, Alexander G. Murley, Catarina Rua, Matilde M. Vaghi, Tara L. White, Guy B. Williams, Trevor W. Robbins

**Affiliations:** a Departments of Psychiatry, Psychology, and Clinical Neurosciences, University of Cambridge, Cambridge, United Kingdom; b Wolfson Brain Imaging Centre, University of Cambridge, Cambridge, United Kingdom; c Department of Psychology, Stanford University, Stanford, California; d Department of Behavioral and Social Sciences, Brown University, Providence, Rhode Island

**Keywords:** 7T magnetic resonance spectroscopy, Cocaine use disorder, Contingency degradation, Creature of Habit Scale, Glutamate-to-glutamine ratio

## Abstract

**Background:**

The balance between goal-directed behavior and habits has been hypothesized to be biased toward the latter in individuals with cocaine use disorder (CUD), suggesting possible neurochemical changes in the putamen, which may contribute to their compulsive behavior.

**Methods:**

We assessed habitual behavior in 48 patients with CUD and 42 healthy control participants using a contingency degradation paradigm and the Creature of Habit Scale. In a subgroup of this sample (CUD: *n* = 21; control participants: *n* = 22), we also measured glutamate and glutamine concentrations in the left putamen using ultra-high-field (7T) magnetic resonance spectroscopy. We hypothesized that increased habitual tendencies in patients with CUD would be associated with abnormal glutamatergic metabolites in the putamen.

**Results:**

Compared with their non–drug-using peers, patients with CUD exhibited greater habitual tendencies during contingency degradation, which correlated with increased levels of self-reported daily habits. We further identified a significant reduction in glutamate concentration and glutamate turnover (glutamate-to-glutamine ratio) in the putamen in patients with CUD, which was significantly related to the level of self-reported daily habits.

**Conclusions:**

Patients with CUD exhibit enhanced habitual behavior, as assessed both by questionnaire and by a laboratory paradigm of contingency degradation. This automatic habitual tendency is related to a reduced glutamate turnover in the putamen, suggesting a dysregulation of habits caused by chronic cocaine use.

Drug addiction is widely regarded as a chronically relapsing disorder, characterized by persistent drug- seeking despite the harm it causes and the declining pleasure gained from drug use (1). This behavioral profile plausibly reflects dysregulation between goal-directed actions [subserved by ventromedial frontostriatal, especially caudate, circuits (2)] and stimulus-driven habits (underpinned by premotor to posterior putamen corticostriatal loops). Growing evidence suggests that stimulant drug exposure causes neuroplasticity in corticostriatal circuits implicated in distinct associative learning mechanisms, leading to impaired action-outcome learning (3), narrowing of goals (4), and enhanced stimulus-driven habits (5, 6, 7). There is evidence of NMDA receptor involvement in the cortico-dorsolateral striatum of rats in habit learning (10, 8, 9), and behavioral training enhances glutamatergic neurotransmission in the same region (11), which is homologous with the human putamen. Drug-induced changes in glutamatergic inputs to the dorsolateral striatum have been linked with the development of automatic habits that persist even in the face of negative consequences (12,13). At present, it is still unclear how the preclinical evidence translates to humans, i.e., whether patients with cocaine use disorder (CUD) exhibit increased habitual behaviors and whether these are related to glutamatergic abnormalities in the human putamen, a key region implicated in habit formation (2,14).

A predominance of the habit system can be tested experimentally by manipulations that either render goal-directed actions meaningless (i.e., by disrupting the perceived causal relationship or contingency between the action and the outcome) or make the outcome undesirable (i.e., by devaluing the outcome). As habits are affected by neither manipulation, individuals with a strong habit system would continue responding irrespective of these manipulations. We have recently shown that appetitive instrumental performance in patients with CUD was indeed unaffected by outcome devaluation, pointing toward strengthening of the appetitive habit system (15). However, patients with CUD in this study also showed significant deficits in reward-based learning (16), suggesting impairments in reinforcement learning or a lack of motivation, which may invalidate the devaluation test. Thus, further evidence is needed to confirm an appetitive habit bias in patients with CUD using an experimental paradigm that does not manipulate outcome value, such as contingency degradation.

If CUD is associated with increased habit formation, one would expect this to be reflected also in patients’ daily habits. Contrary to experimentally induced habits, habitual responses in daily life have often been practiced over prolonged periods. Although these behaviors may have initially been goal directed, through repetition they become autonomous of the goal, so that entering the associated environment is sufficient to trigger the behavior. The Creature of Habit Scale (COHS) (17,18) measures aspects of these daily habits, both involuntary actions triggered by certain environments (automaticity) and sequential actions led by order and regularity (routines).

Habits are subserved by different networks from goal-directed actions, involving sensorimotor regions of the striatum (putamen) and connected sensory and motor cortices (19). Specifically, the putamen has been shown to play a critical role in the automatization of behavior and habit formation (14). While dopamine plays a key role during acute drug exposure, neuroadaptive changes in the glutamate system may be critical for the development of compulsive drug seeking (20). There is growing preclinical evidence suggesting cocaine-induced alterations in other neurotransmitter systems, including glutamate (21,22) and GABA (gamma-aminobutyric acid) (23,24), which may underlie the formation of maladaptive habits (25). There appears to be conflicting evidence on changes of these neurotransmitters in cocaine-addicted humans. While some studies suggest either increased (26) or decreased (27,28) cortical levels, subcortical levels of glutamate were either unchanged (29) or reduced (30). These inconsistencies may result from interactions between dopamine and glutamate (31), possibly reflecting individual differences in striatal dopamine depletion due to varying degrees of cocaine use in the samples studied (31). Moreover, glutamate, glutamine, and GABA levels are difficult to differentiate using magnetic resonance spectroscopy (MRS) at lower field strengths (1.5T or 3T) because of their overlapping spectra (32,33).

The aim of this study was threefold: 1) to provide complementary evidence for increased habit formation in patients with CUD using a contingency degradation paradigm, which, to the best of our knowledge, has not yet been used in humans with CUD; 2) to evaluate the relationship between experimentally induced habits and self-reported habitual tendencies; and 3) to both quantify glutamate, glutamine, and GABA concentration in the putamen using ultra-high-field MRS and establish their relationship with habitual tendencies. We hypothesized that patients with CUD would show increased habitual tendencies, as measured both objectively by a contingency degradation paradigm and by self-report. We further hypothesized that increased habitual tendencies are associated with altered concentrations of glutamate and GABA metabolites in the putamen.

## Methods and Materials

### Participants

In light of the predominance of male cocaine users (34), we recruited 90 men from the community by advertisement and word of mouth. A total of 48 individuals had a history of chronic cocaine use, satisfying the DSM-5 (1) criteria for moderate/severe CUD, whereas the remaining 42 individuals were healthy and without a personal history of substance use disorder (see Table 1 for participant characteristics). Exclusion criteria for all volunteers included a lifetime history of a psychotic disorder, neurological illness or traumatic head injury, and insufficient proficiency in English. All participants were screened for current psychiatric disorders using the Mini-International Neuropsychiatric Inventory (35) and completed the COHS (17,18) to measure routine and automatic behaviors and the Obsessive-Compulsive Inventory-Revised (36) to measure compulsive tendencies. Before testing, all participants were breathalyzed to confirm sobriety and urine samples were tested for undeclared drugs; all samples provided by control participants were drug negative, whereas all samples provided by patients with CUD tested positive for cocaine. All participants provided written informed consent and received monetary compensation for their participation in the study, as approved by the Cambridge Research Ethics Committee.

**Table 1:** Demographics, Personality Traits, and Drug Use Data of All Participants and Selectively of Participants Who Underwent MRS Scanning

Demographics	Control Group	Cocaine Group	Group Comparison	
			*t*_90_, Fisher’s Exact	*p* Value
Behavioral Data				
Sample size, *n*	42	48	–	–
Age, years	40.2 ± 12.5	40.4 ± 9.1	−0.1	.937
Handedness, right/left/ambidextrous, *n*	38/3/1	40/7/1	1.4	.661
Education, years	15.7 ± 2.7	11.0 ± 1.5	10.3	<.001
Routine behavior, COHS score	54.5 ± 10.3	54.3 ± 9.8	0.1	.913
Automaticity, COHS score	30.3 ± 7.3	39.2 ± 7.0	−5.9	<.001
Compulsivity, OCI-R score	8.8 ± 6.0	18.4 ± 11.7	−5.0	<.001
Alcohol use, AUDIT score	3.4 ± 1.7	4.3 ± 5.8	−0.9	.353
Drug use, DAST-20 score	0.1 ± 0.3	–	–	–
Neuroimaging Data				
Sample size, *n*	22	21	–	–
Age, years	38.4 ± 11.2	41.5 ± 10.6	−0.95	.347
Handedness, right/left/ambidextrous, *n*	19/2/1	17/3/1	0.6	.778
Education, years	15.9 ± 2.6	11.0 ± 1.5	−1.8	<.001
Routine behavior, COHS score	56.5 ± 9.7	55.4 ± 10.8	0.3	.734
Automaticity, COHS score	31.0 ± 8.1	41.5 ± 7.3	−4.4	<.001
Compulsivity, OCI-R score	7.4 ± 5.6	17.7 ± 11.1	−3.8	<.001
Alcohol use, AUDIT score	2.9 ± 1.6	4.8 ± 6.1	−1.3	.191
Drug use, DAST-20 score	0.0 ± 0.2	–	–	–

Values are presented as mean ± SD except where noted.

AUDIT, Alcohol Use Disorders Identification Test; COHS, Creature of Habit Scale; DAST-20, 20-item Drug Abuse Screening Test; MRS, magnetic resonance spectroscopy; OCI-R, Obsessive-Compulsive Inventory-Revised.

Patients with CUD had been actively using cocaine for an average of 13 years (SD ±7.7), and most (87%) were using the drug on a daily basis. Patients with CUD reported moderate-to-high levels of cocaine-related compulsivity [Obsessive-Compulsive Drug Use Scale (37), mean (SD) = 33.9 (±10.0)]. None of the healthy volunteers satisfied criteria for substance use disorder, nor were they taking prescribed or illicit drugs on a regular basis, as reflected by low scores on the Alcohol Use Disorders Identification Test (38) and 20-item Drug Abuse Screening Test (39) and drug-negative urine screens on the testing day.

### Behavioral Measures

**Figure 1:**
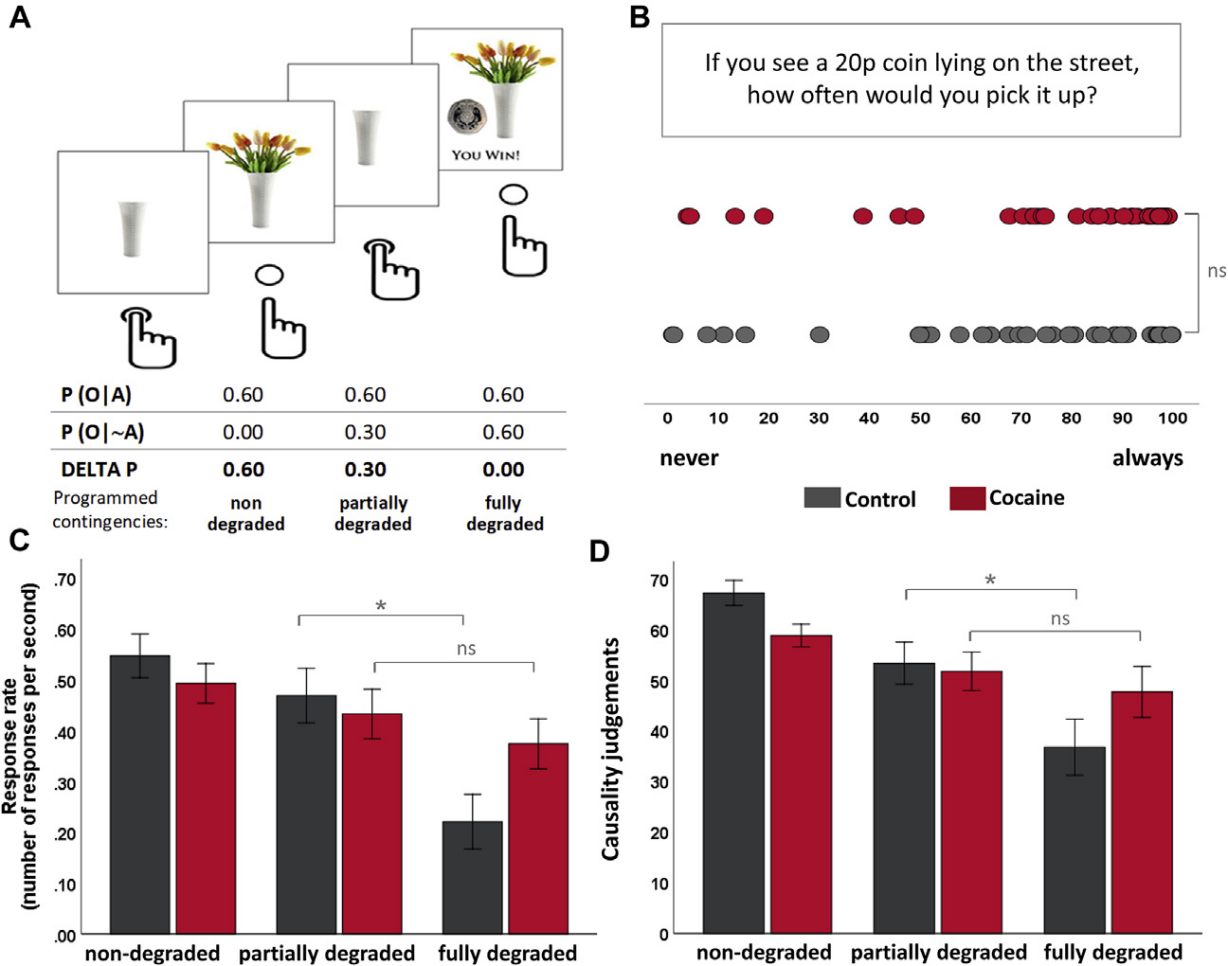
**(A)** Experimental design of a varying degradation of contingency. Participants are shown a white vase on the computer screen, which fills with flowers when the subject presses a dedicated button on the keyboard. Button presses are always associated with a 60% probability of receiving 20 pence. Depending on the experimental condition, not pressing the button was also associated with receiving 20 pence. **(B)** Subjective value of monetary reward. Participants rated on a visual analog scale (0 = never, 100 = always) how likely it was that they would pick up a 20-pence coin lying on the street. The groups did not differ in terms of their subjective value of 20 pence. **(C)** Response rate (number of button presses per second) across the three different task conditions (nondegraded, partially degraded, and fully degraded). Control participants demonstrated sensitivity to changes in the action-outcome contingencies as they significantly reduced their response rate in the fully degraded condition, whereas patients with cocaine use disorder did not. Their response rates between the partially and fully degraded conditions did not differ. **(D)** After each condition, participants were asked to indicate how likely they think it is that pressing the button wins them money. While control participants were aware about the changes in the action-outcome relationship and adjusted their behavior accordingly, patients with cocaine use disorder continued to believe in the effectiveness of their actions to receive a financial reward and so they continued responding accordingly. Error bars denote standard error of the mean. ∗*p* < .05. A, action; ns, not significant; O, outcome; P, probability.

First, we assessed participants’ sensitivity to monetary reward by asking them to rate on a visual analog scale (0 = never, 100 = always) how often they would pick up a 20 pence coin lying on the street (Figure 1B). We then administered a modified version of the contingency degradation task previously used by Vaghi *et al.* (40), which consists of 8 blocks of 120 unsignaled, 1-second trials. Participants were presented with a picture of a white vase on the computer screen, which could be filled with flowers by button press. In 60% of trials, the button press also led to a financial reward, as a 20 pence coin and the message *“You win!”* appeared next to the flowers on the screen for 500 ms (Figure 1A). This action-outcome contingency was established over the first 3 blocks (nondegradation)—a duration sufficient in humans to induce habits (41)—before in block 4 the contingency was partially degraded by superimposing free rewards in 30% of trials (partial degradation) and then fully degraded in block 5, when free rewards were provided at the same rate as action-contingent rewards (full degradation). In block 6, the initial action-outcome contingencies of 60% were reinstated before they were partially degraded in block 7 and fully degraded in block 8. An overview of the conditions and contingencies is shown in Table 2. Participants were informed that sometimes when they press the button they will win money, but at other times nothing will happen. After each block, participants were asked to indicate on a continuous scale how likely their actions were associated with a reward (0 = never, 100 = always). Key outcome variables were the response rate per condition and the causality judgments of button presses leading to rewards.

**Table 2:** Overview of the Task Design, Depicting the Probabilities (P) of the Action-Outcome Contingencies and the Programmed Contingencies per Condition

Block Number	Condition	P (20p Received Following Action)	P (20p Received Following No Action)	ΔP (Programmed Contingency)
1	Nondegraded	0.60	0.00	0.60
2	Nondegraded	0.60	0.00	0.60
3	Nondegraded	0.60	0.00	0.60
4	Partially degraded	0.60	0.30	0.30
5	Fully degraded	0.60	0.60	0.00
6	Nondegraded	0.60	0.00	0.60
7	Partially degraded	0.60	0.30	0.30
8	Fully degraded	0.60	0.60	0.00

### Neuroimaging Measures

After completion of the behavioral task, half of the sample (22 control, 21 CUD) underwent whole-brain T1-weighted MR and single-voxel proton MRS scanning at the Wolfson Brain Imaging Centre, University of Cambridge (United Kingdom) using a 7T Magnetom-Terra scanner (Siemens, Erlangen, Germany). Only participants without MR contraindications such as metal implants, tattoos, or claustrophobia were invited for the scan, but they did not differ from the rest of the sample on any demographic or behavioral variables (Table S1). The scanner was equipped with a single-channel transmit, 32-channel receive array head coil (Nova Medical, Carson, CA).

T1-weighted images were acquired with a 0.75-mm isotropic resolution three-dimensional 2-image magnetization prepared rapid acquisition gradient-echo (3D-MP2RAGE) sequence (42) (echo time/repetition time = 1.99/4300 ms, inversion time = 840/2370 ms, flip angles = 5/6°, acceleration factor = 3, bandwidth = 250 Hz/px, total acquisition time = 8 min 50 s). A 16 × 16 × 35 mm^3^ voxel was placed manually over the left putamen using anatomical landmarks (Figure S1), as this region has previously been linked with increased volume in patients with CUD (43, 44, 45, 46). Spectra were acquired using a short-echo semi-LASER sequence (47,48) (repetition time/echo time = 5000/26 ms, 64 repetitions) with FASTESTMAP shimming (49) and with variable power radiofrequency pulses with optimized relaxation delays water suppression calibration (50).

FSL was used to assess the structural MR images. T1 images were brain extracted using the FSL Brain Extraction Tool (51). A study-specific gray matter template was created by iterative nonlinear registration of participants’ gray matter images to the gray matter ICBM-152 template. All images were registered to the study-specific template, modulated using Jacobian warp fields (52), concatenated into a four-dimensional image, and smoothed with a kernel of full-width-half-measure of 3 mm. Mean gray matter volumes for the putamen in each participant were calculated by summing the gray matter values over the relevant segments of the Harvard-Oxford atlas.

The 64 individual spectral transients from each participant were saved separately and corrected for effects of eddy currents, frequency, and phase shifts using MRspa (Dinesh Deelchand, University of Minnesota, Minneapolis, MN; www.cmrr.umn.edu/downloads/mrspa). Metabolites between 0.5 and 4.2 parts per million (including glutamate, glutamine, and GABA) were quantified using LCModel (version 6.2-3) (53) with water scaling and a simulated basis set that included experimentally acquired macromolecule spectra. Molecules were water scaled using unsuppressed water spectra acquired before and after the 64 repetitions, assuming no cerebrospinal fluid content in the voxel.

### Statistical Analysis

Demographic and behavioral data were analyzed using SPSS version 25 (IBM Corp., Armonk, NY). Differences between conditions were analyzed using repeated-measures analysis of covariance models with level of contingency degradation (none/partial/full) as the within-subject factor and group (control/CUD) as the between-subject factor. Mean years of education were included as a covariate to control for differences in educational achievements between the groups. Where assumptions of heterogeneity of covariance were violated, degrees of freedom were corrected using the Greenhouse-Geisser approach. Group differences of questionnaire data, rating scores, and metabolite concentrations were determined using univariate and multivariate analysis of variance models, respectively. All statistical tests were two-tailed and the significance level was set at .05.

## Results

### Demographics and Questionnaire Data

**Figure 2:**
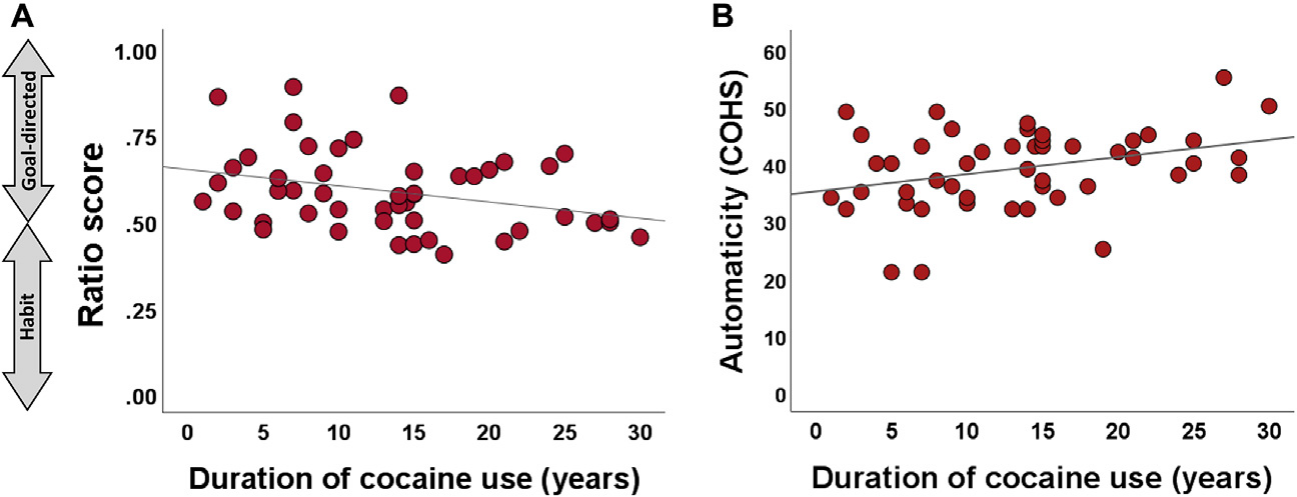
We calculated a ratio score of pairs of nondegraded and partially or fully degraded blocks to estimate the balance between the goal-directed and habitual systems, as described by Vaghi *et al.* (40). For each pair, the number of responses in the nondegraded block was divided by the sum of responses in both the contingent and the degraded blocks. Values close to 1 reflect high sensitivity to the reinforcement contingency (indicative of goal-directed tendencies) and values close to 0.5 reflect a similar response patterns of the contingent and degraded conditions (indicative of habitual tendencies). **(A)** In the fully degraded condition, patients with cocaine use disorder showed increased habitual responses the longer they have been using cocaine (*r* = −3.1*, p* < .05). **(B)** The longer the duration of cocaine use, the more automatic habits patients with cocaine use disorder reported on the COHS (*r* = .33*, p* < .05). COHS, Creature of Habit Scale.

As shown in Table 1, the groups were matched on age, handedness, and sensitivity to the 20 pence reward value, but patients with CUD had spent less time in full-time education. As years of education were correlated with the response latencies (*r* = .463, *p* = .002), they were included as a covariate in the analysis. In terms of habitual tendencies (as measured by COHS), patients with CUD reported to engage in daily routines to the same degree as control participants (*F*_1,88_ = 0.01, *p* = .913) but scored significantly higher on automaticity than control participants (*F*_1,88_ = 54.5, *p* < .001) the longer they had been using cocaine (*r* = .33, *p* = .02) (Figure 2B). Pathological habits, as measured by the Obsessive-Compulsive Inventory-Revised scale, were also increased in patients with CUD compared with control participants (*t*_72.09_ = −4.97, *p* < .001).

### Behavioral Results

In line with our principal behavioral prediction, we found a significant main effect of contingency degradation on response rate (*F*_1.6,138.5_ = 5.1, *p* = .012), indicating that both groups were able to respond according to the action-outcome association (see also Figure S2). Although response rates declined following contingency degrading, the overall decline was less steep in patients with CUD than in control participants, as reflected by a significant group-by-contingency interaction (*F*_1.6,138.5_ = 4.9*, p* = .014). As shown in Figure 1C, control participants reduced their responses as a function of degradation, but patients with CUD did not. Furthermore, the increased response rate in the fully degraded condition of patients with CUD was associated with the duration of cocaine use (*r* = −3.1, *p* = .042) (Figure 2A). Notably, response rates between the nondegraded and partially degraded conditions did not differ between the groups (*F*_1,87_ = 0.6*, p* = .813). This response pattern was also mirrored by the group-by-contingency interaction in participants’ causality judgments (*F*_1.6,140_ = 3.3, *p* = .048) (Figure 1D), suggesting that even on the fully degraded trials, patients with CUD were following their perceived causal beliefs. Indeed, participants’ response rates and their subjective awareness of causality were highly correlated (partially degraded: *r* = .37, *p* < .001; fully degraded: *r* = .51, *p* < .001).

**Figure 3:**
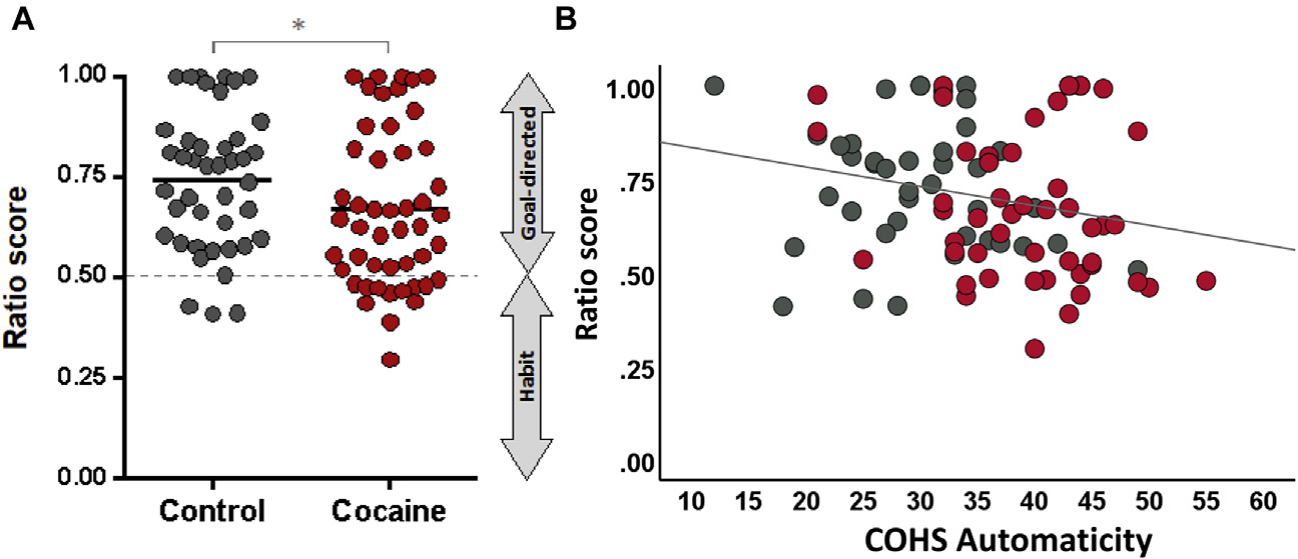
Goal-to-habit ratio score for the condition in which the action-outcome relationship was fully degraded. The score was computed from the number of responses during the nondegraded condition divided by the sum of responses in the fully degraded and nondegraded conditions. Values close to 1 suggest high sensitivity to the reinforcement contingency (indicative of the goal-directed system) and values close to 0.5 suggest that the response pattern does not differentiate between nondegraded and degraded conditions (indicative of the habit system). **(A)** When the action-outcome contingency was fully degraded, patients with cocaine use disorder exhibited a less goal-directed response tendency than healthy control participants. **(B)** COHS automaticity scores (i.e., the degree to which behavioral responses are involuntarily triggered by specific contexts) were associated with more habitual response tendencies on fully degraded trials (*r* = −.23*, p* < .05; control participants: *r* = .12, *p* = .445, cocaine use disorder: *r* = .26, *p* = .069). COHS, Creature of Habit Scale.

We also calculated a ratio score for the two conditions (partially degraded and fully degraded contingencies) to test our hypothesis of strong habitual control in CUD patients. As shown in Figure 3A, in the fully degraded condition, the ratio scores differed significantly between the groups (*F*_1,87_ = 4.8, *p* = .031), as a quarter of patients with CUD (25%) exhibited a habitual strategy (i.e., a ratio value ≤ 0.5) compared with 7% of the control group (Fisher’s exact *p* = .026). This goal-to-habit ratio correlated with participants’ self-reported automaticity (*r* = −.23*, p* = .029) (Figure 3B) but not with obsessive-compulsive behavior (*r* = −.01, *p* = .371). The ratio scores of the partially degraded condition were not significantly different between the two groups (*F*_1,87_ = 0.02, *p* = .898).

### Neuroimaging Results

**Figure 4:**
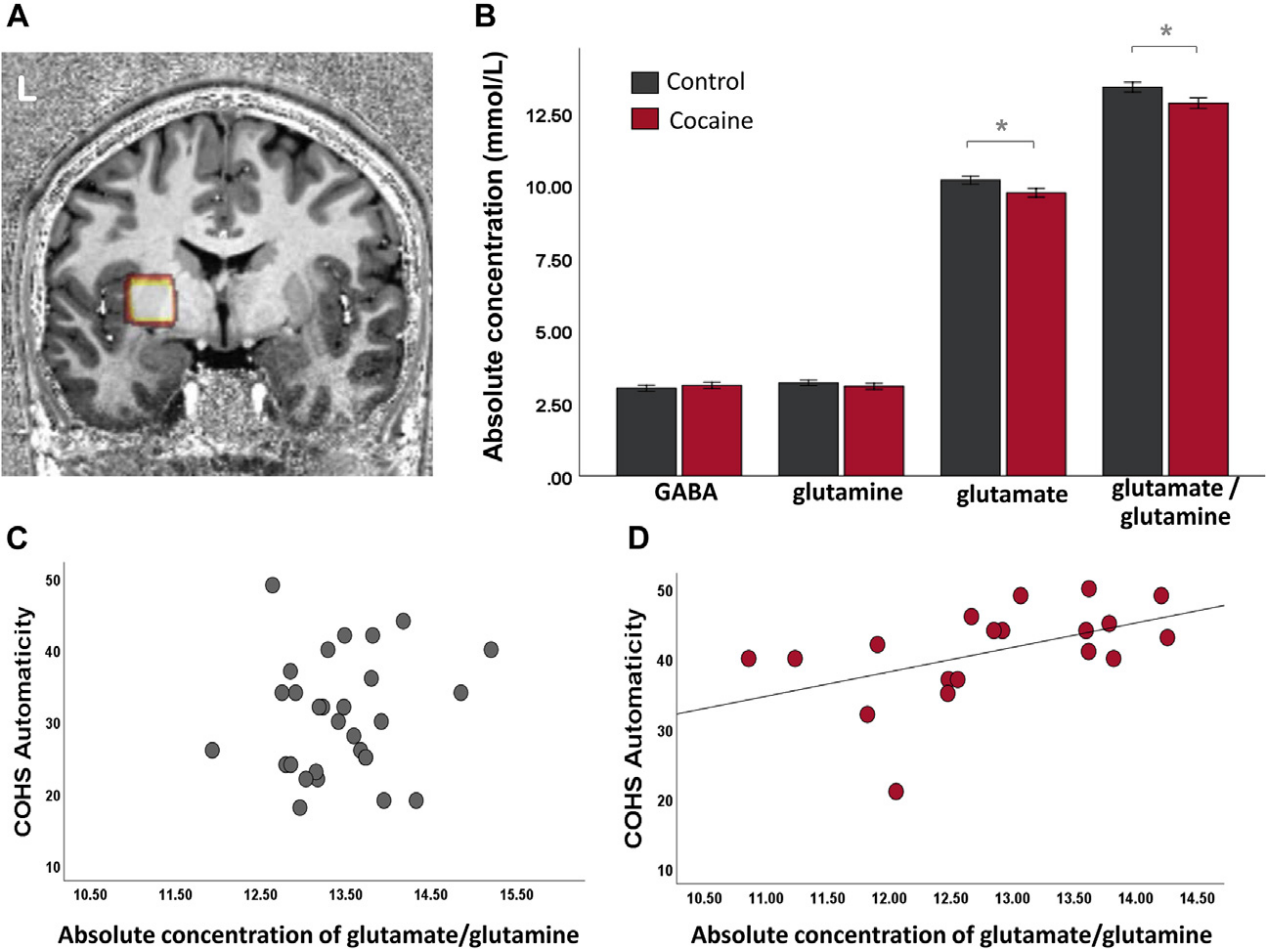
**(A)** Voxel placement and representative spectra from the left putamen. **(B)** Mean GABA (*t*_39_ = −0.66, *p* = .514; *d* = 0.21), glutamine (*t*_39_ = 0.79, *p* = .437; *d* = 0.24), and glutamate (*t*_39_ = 2.14, *p* = .039; *d* = 0.64) concentration in the left putamen, as well as the glutamate-to-glutamine ratio (*t*_39_ = 2.11, *p* = .33; *d* = 0.69) in both healthy control participants and patients with cocaine use disorder (error bars denote standard error of the mean; ∗*p* < .05). **(C)** Self-reported Creature of Habit traits (COHS automaticity levels) are not associated with the glutamate-to-glutamine ratio in the left putamen in control participants (*r* = .11, *p* > .5). **(D)** In patients with cocaine use disorder, however, there is a strong relationship between COHS automaticity and the glutamate-to-glutamine ratio measured in the left putamen (*r* = .49, *p* < .05). COHS, Creature of Habit Scale; GABA, gamma-aminobutyric acid.

The demographics and questionnaire scores of the two subgroups undergoing MR scanning are shown in Table 1. The groups did not differ on putamen volume (*t*_41_ = 0.40, *p* = .692), which is why we did not include volume as a covariate in the analysis. Two MRS spectra of patients with CUD were excluded because of poor quality. As shown in Figure 4D, the groups differed significantly in glutamate concentration (*F*_1,39_ = 4.6, *p* = .039; *d* = 0.64) and the glutamate-to-glutamine ratio (*F*_1,39_ = 4.9, *p =* .033; *d* = 0.69). Concentrations of glutamine (*F*_1,39_ = 0.6, *p* = .437; *d* = 0.24) and GABA (*F*_1,39_ = 0.4*, p* = .514; *d* = 0.21) were not significantly different between groups. These results were not explained by differences in scan quality; water linewidth, signal to noise, and metabolite Cramér-Rao lower bound were not different between the two groups (Table S2). The glutamate-to-glutamine ratio correlated with self-reported automaticity in both groups; i.e., higher automaticity levels in patients with CUD were associated with a lower glutamate-to-glutamine ratio (*r* = .5, *p* = .034) (Figure 4B); this relationship was not seen in the control group (*r* = .1, *p* = .623) (Figure 4A), but the correlation strength did not significantly differ between the groups (*z* = −1.25, *p* = .106). There was also no relationship between these metabolites and contingency degradation performance, the duration of cocaine use, or the number of tobacco cigarettes smoked (all *p* > .1). Putamen volume was not associated with glutamate-to-glutamine ratio, behavioral performance, or self-reported automaticity (all *p* > .1).

## Discussion

A key finding of this study is that patients with CUD had no problems learning action-outcome associations (Figure S2), but they failed to adjust their responses when the relationship between their actions and the consequences was fully degraded. This increased response tendency is indicative of habitual control and was more pronounced the longer patients with CUD had been using cocaine. Although habits have often been considered to be the product of overtraining, and in this respect, the training in this study may appear rather short, recent evidence contradicts the necessity of overtraining (18,41,54). Increased habitual tendencies were also reflected by levels of automatic behavior patterns in their daily lives reported by patients with CUD, which were most evident the longer they had been using cocaine. These automatic behavioral patterns were associated with reduced glutamate turnover in the putamen, a key region implicated in habit formation. Our results provide compelling evidence for increased habitual tendencies in CUD and show, to the best of our knowledge, for the first time a link in humans between increased habitual tendencies and abnormal glutamate metabolites, indicating possibly altered glutamate neurotransmission in the habit pathway in patients with CUD.

### Causal Beliefs and Instrumental Actions in CUD Unaffected by Full Contingency Degradation

Consistent with our hypothesis, we observed in patients with CUD greater habitual responding during contingency degradation than their non–drug-using peers. As shown in Figure 1C, in the nondegraded condition, all participants performed the instrumental action (pressing the button to obtain a financial reward), suggesting that they established a causal representation between their performance and the rewarding outcome (Figure 1D). Only when the action-outcome contingency was completely decoupled, i.e., when rewards became available irrespective of instrumental actions, did the response rate in control participants decline significantly, as well as their beliefs in the consequences of their actions. In patients with CUD, however, instrumental performance was insensitive to this manipulation, as they appeared unable to update the previously learned action-outcome association and thus continued to believe in the effectiveness of their actions. Their unfailing belief in their actions cannot be attributed to a misunderstanding of the task but reflects their lack of awareness of the disruption of the established action-outcome contingency. This is indicative of habitual control (55) and may suggest that the goal-directed system of patients with CUD was no longer in charge of their behavior but had deferred control to the habit system. A similar, albeit not identical, profile has also been observed in patients with obsessive-compulsive disorder (40), further supporting the notion of a habit bias being characteristic for disorders of compulsion. Moreover, the relationship between the continued responses of patients with CUD following the breakdown of the action-outcome association and the duration of their cocaine use concurs with a large body of preclinical research indicating that stimulant drugs facilitate habit formation (6,7,12,56).

Partial degradation of the action-outcome contingency was, in both groups, insufficient to change their causal beliefs and their instrumental actions significantly. Given that contingency information is calculated through the overall probability of an action producing an outcome (57,58), it is possible that the higher likelihood of obtaining a reward following the instrumental action than following a nonaction led participants not to change their behavior significantly. Only when this contingency was completely disrupted did healthy individuals adjust their behavior and beliefs accordingly (40,59, 60, 61), but patients with CUD failed to do so (Figure 1).

### Putative Neural Correlates of Contingency Degradation Performance

Sensitivity to action-outcome contingencies involves a number of brain regions including the ventromedial prefrontal cortex for encoding outcome value (62,63), the caudate nucleus and perigenual anterior cingulate cortex for detecting the causal relationship between actions and outcomes (64, 65, 66), and the dorsal hippocampus for encoding the instrumental contingency (67). The dorsolateral putamen has been shown to prevent regular updates about contingency information, thereby facilitating the formation of habits (63,68,69) and supporting the hypothesis that a strong habit system relies on the putamen (70,71). Here, we found no evidence that changes in the putamen are implicated in contingency degradation performance, but the impairment of patients with CUD in detecting changes in the action-outcome relationship and their unawareness of these changes may point toward deficits in the perigenual anterior cingulate cortex (64). Prior research has shown that ventromedial prefrontal cortex lesions impair individuals’ ability to establish awareness about causal action-outcome contingencies, but these deficits alone are insufficient to impair participants’ ability to adjust their behavior when these contingencies break down (60).

### Abnormal Glutamate Turnover Predicts Self-reported Habits in Patients With CUD

In keeping with our previous work suggesting modulatory effects of stimulant drug exposure on automatic, environmentally triggered behavior (17), patients with CUD in this study reported engaging in such behavior to a significantly greater extent than their non–drug-using peers. Moreover, the high levels of automaticity of patients with CUD were significantly correlated not only with their duration of cocaine use but also with their reduced glutamate-to-glutamine ratio in the putamen (Figure 4B). This ratio may reflect the operation of a key mechanism to regulate the concentration of extracellular glutamate that protects the brain from overexcitation. Thus, whenever glutamate is released by neurons during neurotransmission, it is rapidly taken up by surrounding astroglia and converted into its inactive form of glutamine before it is released again into the synaptic cleft and taken up by neurons to be synthesized into glutamate (72). Stimulant drugs have been shown to acutely increase glutamate levels and turnover (12,73), but regular drug use impairs glutamate homeostasis by altering glial-neuronal interactions (74). The glutamate-to-glutamine ratio is therefore an important indicator, reflecting not only glutamate turnover rate but also the activity of glutamate-releasing neurons (75). The reduced glutamate-to-glutamine cycle, as seen in our patients with CUD, may thus point toward an impaired neuron-astrocyte crosstalk, which leads to diminished glutamate turnover in the putamen and may facilitate automated habits (Figure 4).

The significant reduction in glutamate concentration in the putamen and the low glutamate turnover in patients with CUD is in keeping with previously published findings in CUD, suggesting a substantial downregulation of glutamate neurotransmission, including widespread reduction in glutamate receptors (29,76,77) and low glutamate concentrations (27,30). To the best of our knowledge, our study is the first to show in humans a link between abnormal glutamate turnover and increased automatic habits. We acknowledge that our data may be at odds with preclinical research in two respects: 1) that enhanced corticodorsolateral striatal glutamate neurotransmission is associated with habit learning (11) and 2) that stimulant drugs acutely increase rather than decrease glutamate signaling in the putamen (12), which has been linked with synaptic reorganization and accelerated formation of habits (12). However, long-term cocaine use downregulates both the dopamine (78) and glutamate systems (29), which interact to produce reduced striatal glutamate concentration (31), as seen here. These changes likely impair the regulation of habits by top-down mechanisms of inhibitory control (79), normally mediated by excitatory frontostriatal projections (79,80).

Despite the significant reduction in the glutamate-to-glutamine ratio in patients with CUD, their glutamine levels were not measurably different from control participants (Figure 4). Glutamine serves as a precursor not only for glutamate but also for GABA (81), which was not measurably altered in the putamen of patients with CUD. The selective decline in glutamate concentration may suggest cocaine-induced impairments in glutamate synthesis, possibly through expression and activity of the enzyme glutaminase (82).

Preclinical studies show that the glutamate transporter is generally downregulated in the striatum, at least following exposure to prolonged access to cocaine (excepting the nucleus accumbens shell region) (83,84), and that fewer synapses are unsheathed by astroglia, although this has only to date been investigated in the nucleus accumbens core (85). Moreover, restoring glutamate homeostasis with *N*-acetylcysteine can recover goal-directed from habitual behavior (56) and rescue cocaine-induced reductions in GLT-1 proteins resulting from intravenous cocaine self-administration in the dorsolateral striatum as well as the nucleus accumbens core (86).

### Strengths, Weaknesses, and Wider Implications

The strengths of the study include the assessment of habits both objectively using a contingency degradation paradigm and subjectively by self-report in a large sample and the significant correlation between the two (Figure 3B). We successfully circumvented potential shortcomings associated with the use of outcome devaluation paradigms in CUD by manipulating the action-outcome contingency rather than the outcome, which is especially difficult to manipulate for outcomes such as drugs and generally works best for outcomes such as food. However, both paradigms, contingency degradation and outcome devaluation, are widely considered the gold standard of testing habits, and our findings are consistent with prior work (4,7,15,17,56). Other paradigms, such as the two-stage decision-making task or pavlovian-to-instrumental transfer procedures, may not be comparable, thus explaining some apparent inconsistencies in the literature. For example, increased habitual responding following outcome devaluation has been reported both in healthy individuals under the acute influence of alcohol (87) and in patients with alcohol use disorder (63) but not using the two-stage decision-making task (88) or a pavlovian-to-instrumental transfer paradigm (89). A variety of measures and experimental paradigms is thus warranted to understand the interplay between goal-directed and habitual control in health and disease.

Metabolic measurements were obtained at ultra-high-field strength (7T), which allowed us to measure concentrations separately from their precursors, i.e., providing information not only about glutamate turnover but also about glutamate synthesis. The inclusion in the study of just male patients with CUD who were actively using cocaine may limit the generalizability of our findings to women with CUD and individuals in recovery. Limitations further include the lack of functional brain data such as resting-state or task-related activation to evaluate the functional implications of glutamate concentration and the restriction of metabolic measurements to just the putamen. We are also unable to determine the precise cortical (or thalamic) origin of the apparent reduction of glutamate transmission. In light of the performance of patients with CUD during contingency degradation—a task that requires monitoring of reinforcement contingencies—measuring glutamate concentration in the caudate nucleus and perigenual anterior cingulate cortex would be of particular interest. It is possible, for example, that a general reduction in glutamate transmission in the caudate may impair goal-directed behavior, which would then indirectly increase habitual control (55). Our findings may have possible therapeutic implications, prior work having shown that *N*-acetylcysteine normalizes glutamate concentrations in the dorsal anterior cingulate cortex (26) and can remediate relapse to drug taking in rodents (17). However, whether it could restore the balance between goal-directed and habitual behavior is as yet unclear.

These results add to the growing evidence indicating that cocaine addiction has profound effects on corticostriatal glutamate neurotransmission (90) associated with enhanced habitual tendencies, which may exacerbate compulsive drug-seeking behaviors.

## Supplementary Material

Supplementary Materials

Key Resources Table
