# Supplementary material for: Reduced Glutamate Turnover in the Putamen Is Linked With Automatic Habits in Human Cocaine Addiction
Source: Biol Psychiatry. 2021 May 15;89(10):970–9. doi: 10.1016/j.biopsych.2020.12.009 (PMC8083107; doi:10.1016/j.biopsych.2020.12.009)
Supplement: Supplementary Materials [file mmc1.pdf]

Reduced Glutamate Turnover in the Putamen Is Linked With Automatic Habits in Human Cocaine Addiction

Supplementary Information

Karen D Ersche, Tsen Vei Lim, Alexander G Murley, Catarina Rua,
Matilde M Vaghi, Tara L White, Guy B Williams, Trevor W Robbins

Supplementary Methods

Participants

Twenty-three CUD patients also met DSM-5 criteria for opiate use disorder, eight for cannabis use disorder and three for alcohol use disorder. All CUD patients were smoking tobacco regularly, 15 were prescribed methadone (mean dose 48.0mg, SD±13.9) and eight were prescribed buprenorphine (mean dose 7.5mg, SD±3.5). Other relevant prescribed medication include analgesia (n=7), benzodiazepines (n=4), and antidepressants (n=6). Four controls reported occasional tobacco smoking.

Statistical Analysis

In preparation for parametric analyses, behavioral data were square-root transformed to reduce skew, but untransformed values are displayed in the figures and tables. Mean values of metabolites were imported into SPSS for group comparisons and correlational analysis. Chi-square or Fisher's exact tests were used for the analysis of categorical data.

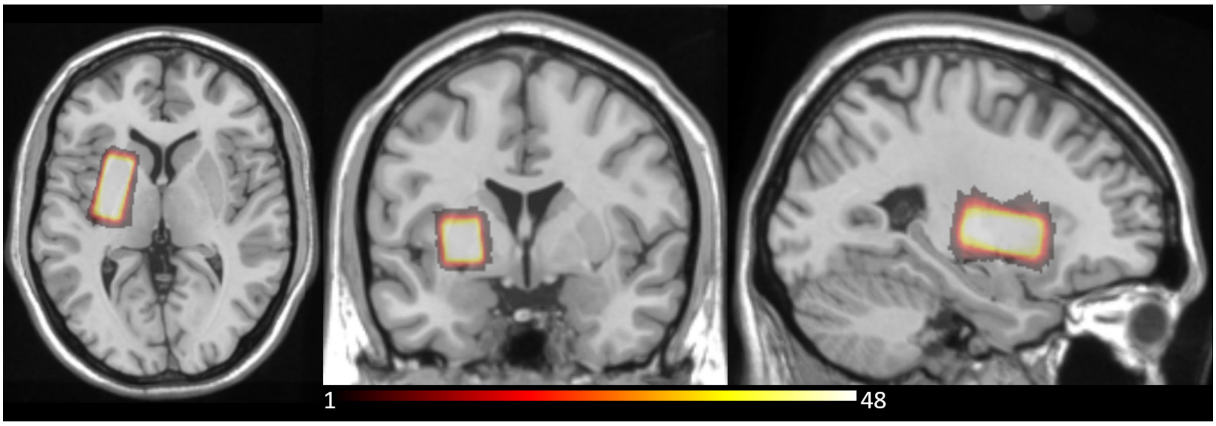

Figure S1: Left putamen voxel placement: Sum of all 43 participant voxels warped and superimposed onto the MNI template brain. The heatmap shows the high degree of overlap between participants (white=overlap of all participants).

Table S1: Demographics, personality traits, drug use and behavioral data [means and standard deviation, (SD) in parentheses] of participants who were invited for MR scanning and those who were not.

	**Scanning Group** (n=43)		**Non-scanning Group** (n=47)		**Group Comparison**	
**Demographics**	**Mean**	**±SD**	**Mean**	**±SD**	**F/t_{90}, Fisher's exact**	***p***
Age (years)	40.6	10.7	39.9	10.9	-.32	0.749
Handedness (left: right: ambidextrous)	36 : 5 : 2		42 : 5 :0		1.96	0.217
Education (years)	12.9	3.1	13.5	3.3	0.85	0.398
Subjective value of 20p (rating)	72.2	30.1	81.3	25.5	-1.55	0.126
Routine behavior (COHS score)	53.0	9.7	55.9	10.2	1.40	0.166
Automaticity (COHS score)	34.0	7.4	36.1	9.3	1.19	0.236
Compulsivity (OCI-R score)	15.2	11.0	12.4	10.1	-1.27	0.207
Alcohol use (AUDIT score)	3.9	4.4	3.8	4.5	-0.11	0.914
Drug use (DAST-20 score)	0.1	0.3	0.0	0.2	-0.63	0.532
Response rate (NonDeg)	63.6	11.6	62.4	13.5	1.48	0.228
Response rate (PartDeg)	57.4	16.9	48.7	22.8	2.82	0.097
Response rate (FullDeg)	46.0	27.4	40.0	28.7	1.04	0.312

Table S2: Measures of magnetic resonance spectroscopy quality and voxel tissue composition. [CRLB: Cramer-Rao Lower Bounds; SNR: Signal-to-Noise Ratio]

	Cocaine Group		Control Group		Group Comparison		
	Mean	±SD	Mean	±SD	t-value	df	*p*-value
**Absolute CRLB**[1]							
GABA	24.746	5.048	24.027	2.957	-0.623	46	0.536
Glutamate	18.345	2.992	19.327	3.193	1.068	46	0.291
Glutamine	19.532	2.599	18.949	4.25	-0.591	46	0.558
SNR	51.158	8.348	54.414	7.307	1.427	46	0.160
Linewidth	19.642	3.387	18.16	2.115	-1.87	46	0.068
**Tissue composition** (% of MRS voxel)							
Grey	64.355	9.517	67.735	6.361	1.478	46	0.146
White	32.128	6.38	35.363	9.651	-1.401	46	0.168

(1) Kreis R. The Trouble With Quality Filtering Based on Relative Cramer-Rao Lower Bounds. *Magn Reson Med* 2016;75(1):15-8.

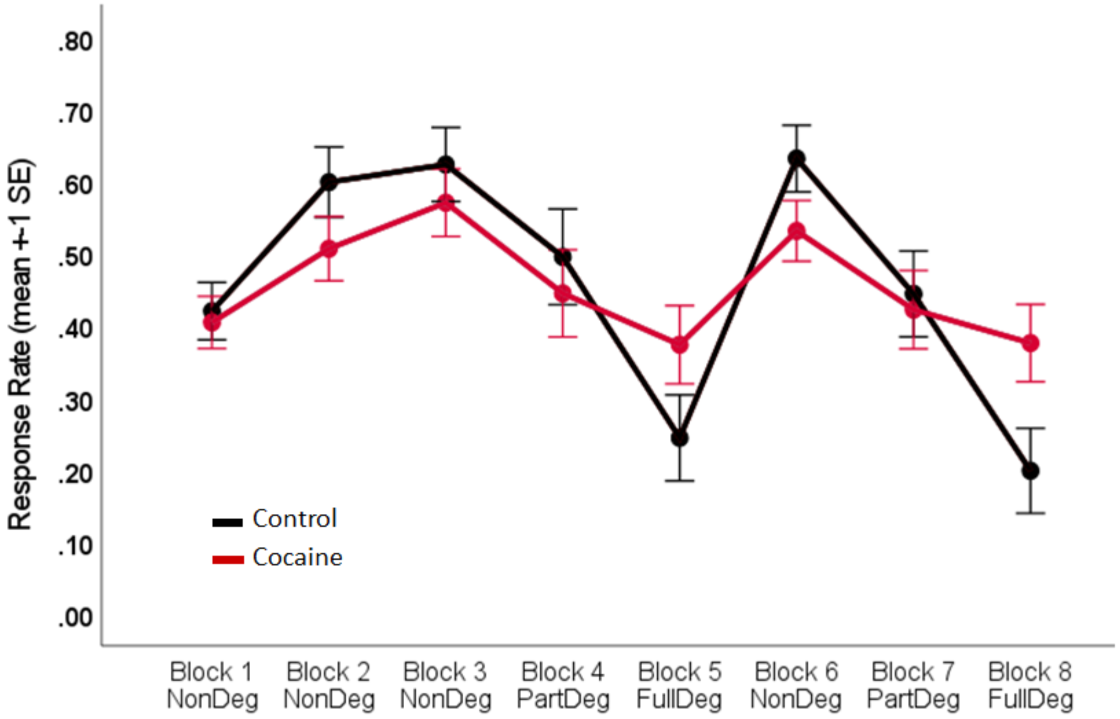

Figure S2: Performance data in the full sample (n=90) over eight blocks and the three experimental conditions: non-degraded (NonDeg), partially degraded (PartDeg), and fully degraded (FullDeg). The increase in the response rate in blocks 1 – 3 suggests that participants learned the action-outcome contingencies, before in block 4 the contingency was partially degraded, and then fully degraded in block 5.

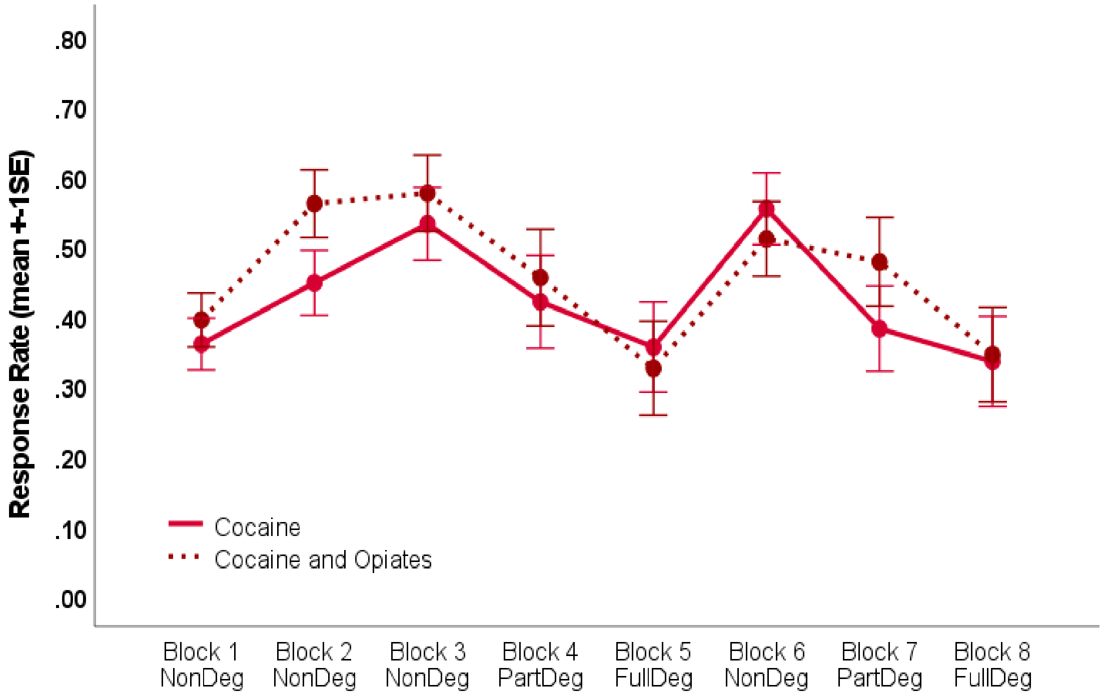

Figure S3: Task performance in CUD subgroups of patients with and without opiate addiction.
